# Geometric Models for Seed Shape Description and Quantification in the Cactaceae

**DOI:** 10.3390/plants10112546

**Published:** 2021-11-22

**Authors:** José Javier Martín-Gómez, Diego Gutiérrez del Pozo, Ángel Tocino, Emilio Cervantes

**Affiliations:** 1 Instituto de Recursos Naturales y Agrobiología del Consejo Superior de Investigaciones Científicas (IRNASA-CSIC), Cordel de Merinas, 40, E-37008 Salamanca, Spain; jjavier.martin@irnasa.csic.es; 2 Herbario Amazónico del Ecuador ECUAMZ, Universidad Estatal Amazónica UEA, Carretera Tena a Puyo Km. 44, Puyo EC-150950, Ecuador; diego.gutierrez.pozo@gmail.com; 3 Departamento de Matemáticas, Facultad de Ciencias, Universidad de Salamanca, Plaza de la Merced 1-4, E-37008 Salamanca, Spain; bacon@usal.es

**Keywords:** Archimedean spiral, cactaceae, geometry, models, morphology, seed shape, spiral

## Abstract

Seed shape in species of the Cactaceae is described by comparison with geometric models. Three new groups of models are presented, two for symmetric seeds, and a third group for asymmetric seeds. The first two groups correspond, respectively, to superellipses and the combined equations of two semi-ellipses. The third group contains models derived from the representation of polar equations of Archimedean spirals that define the shape of asymmetric seeds in genera of different subfamilies. Some of the new models are geometric curves, while others are composed with a part resulting from the average silhouettes of seeds. The application of models to seed shape quantification permits the analysis of variation in seed populations, as well as the comparison of shape between species. The embryos of the Cactaceae are of the peripheral type, strongly curved and in contact with the inner surface of the seed coat. A relationship is found between seed elongation and the models, in which the genera with elongated seeds are represented by models with longer trajectories of the spiral. The analysis of seed shape opens new opportunities for taxonomy and allows quantification of seed shape in species of the Cactaceae.

## 1. Introduction

The Cactaceae Juss., with about 2000 species distributed in ca. 120 genera, are characterized by succulent photosynthetic stems. The family has been included in the order Caryophyllales Juss. ex Bercht. & J.Presl [1], in the suborder Portulacineae Thorne [2] which included Cactaceae, Portulacaceae, Didiereaceae, and Basellaceae, as well as other succulent plant families (Anacampserotaceae, Halophytaceae, Montiaceae, and Talinaceae) [3,4,5]. 

Cacti are common in the arid ecosystems of America, in the southern States of USA and Mexico, north-eastern Brazil, and along the slopes of the Andes from Ecuador to Argentina and Chile. Many species also live in humid forests as epiphytes. All known species are originally American with the only exception of *Rhipsalis baccifera* (J.S. Muell.) Stearn, found also in local populations in tropical Africa and Sri Lanka.

The Cactaceae have been divided into four subfamilies: Cactoideae Eaton, Maihuenioideae P. Fearn, Opuntioideae Burnett, and Pereskioideae Engelm. The Cactoideae and Opuntioideae contain, respectively, 100 and 9 genera [4,5]. *Maihuenia* (Phil.) Britton & Rose, with two cushion-forming, mucilaginous species, is the only genus in the Maihuenioideae. *Pereskia* Mill, together with *Leuenbergeria* Lodé in the Pereskioideae, are woody with large, glossy leaves and long thorns on the stem, and not all are succulents. The Cactoideae, with ten tribes, is the most diverse subfamily of globular and columnar cacti including all the iconic species in American dessert biomes [5]. The Opuntioideae correspond to the prickly pear cacti and relatives, with seeds enclosed by a bony aril that is considered a key diagnostic in this subfamily, except in the genus *Pterocactus*, which possess flattened winged seeds [3].

The flowers of the Cactaceae have interesting peculiarities, such as the presence of the perianth and the pericarpel, the stem-tissue enclosing the inferior ovary [6,7].

Difficulties for taxonomy in this family are partly due to three reasons: (1) A large amount of homoplasy in their characters [8]; (2) The high degree of hybridization [5]; and (3) The abundance of synonyms [9]. Morphological description has been the basis for taxonomy. The identification and classification of cacti has been traditionally based on their flowers and fruits, while seed shape has received comparatively less attention. Diverse aspects of seed morphology have been considered as valuable taxonomic criteria for some genera, such as for example the presence of a keel in *Cereus* [10,11,12], an aril or strophiole in *Blossfeldia* and the Opuntioideae [13,14].

The analysis of seed surface structure by electron microscopy has given useful information in the taxonomy of *Ferocactus* Britton & Rose [15], *Pachycereus* (A. Berger) Britton & Rose [11], *Neobuxbaumia* Backeb. [16], *Stenocereus* (A. Berger) Riccob. [17], and more recently in *Melocactus* Link & Otto [18]. Nevertheless, general descriptions of seed morphology have not been fully applied in taxonomy because the description of seeds has been insufficient and often based on the use of ambiguous or imprecise adjectives.

According to seed shape, Barrios et al. [19] classified the seeds of the Cactaceae in the following six types: 1: globular (*Blossfeldia liliputana*); 2: lenticular (*Pereskia bleo*); 3: pyriform (*Escobaria cubensis*); 4: mussel-shaped (*Selenicereus grandiflorus*); 5: reniform (*Neobuxbaumia multiareolata*); 6: hat-shaped (*Frailea phaeodisca*). This classification is similar to others, see for example [20,21], sharing an important drawback that consists of a poor definition of the terms used, and, in consequence, imprecision in the groups formed. The classification of a seed in a group does not exclude others (globular is not clearly different from lenticular, lenticular is not clearly different from reniform, etc.) and this occurs because there is not a model for each group defined by a geometric figure (a hat, a mussel, and a pear are not geometric objects, and in consequence, they are not precisely defined). To overcome these problems, the morphological description of seeds based on the comparison of seed images of defined orientation with geometric models defined by algebraic equations was proposed [22,23]. Morphological descriptions based on geometric models have been applied to seeds in diverse genera and families [24,25,26,27,28,29,30,31,32,33,34,35,36,37].

Models based on the cardioid or modified cardioids were first applied to the description of seeds of the model plant *Arabidopsis thaliana* (L.) Heynh., the model legumes *Lotus japonicus* L. and *Medicago truncatula* Gaertn., as well as *Capparis spinosa* L., in the Capparaceae and *Rhus tripartita* (Ucria) Grande in the Anacardiaceae [24,25,26,27,28]. The seeds of *Triticum* sp. in the Poaceae and *Ricinus communis* L. and *Jatropha curcas* L. in the Euphorbiaceae were described by the similarity of their images to ellipses of varied x/y ratio [29,30,31]. Among the Ranunculales, oval-shaped seeds occur frequently in the Berberidaceae, Euptelaceae, and Lardizabalaceae while a cardioid shape is more common in the Papaveraceae [32]. The Cucurbitaceae (order Cucurbitales) have characteristic oval-shaped seeds [33].

A series of geometric models have been described also for the Arecaceae and the Vitaceae [34,35]. In the Arecaceae, the seeds correspond to the following models: circular, elliptic, oval, lemniscate, superellipse, cardioid and derivatives, lenses, and waterdrops [34]. Some of these types were also observed in the Vitaceae, in particular lenses, superellipses, and waterdrops, while diverse types of waterdrops and heart curves, obtained by the modification of equations of an ellipse, are characteristic of genera and species in this family. The models obtained were adjusted with precision to the shape of seeds in genera and species of this family [35].

Our objective was to obtain geometric models for the description of seed shape in the Cactaceae. With this basis, seed shape description and quantification could be applied in taxonomy.

## 2. Results

### 2.1. New Geometric Models Based on the Equation of an Ellipse

**Figure 1:**
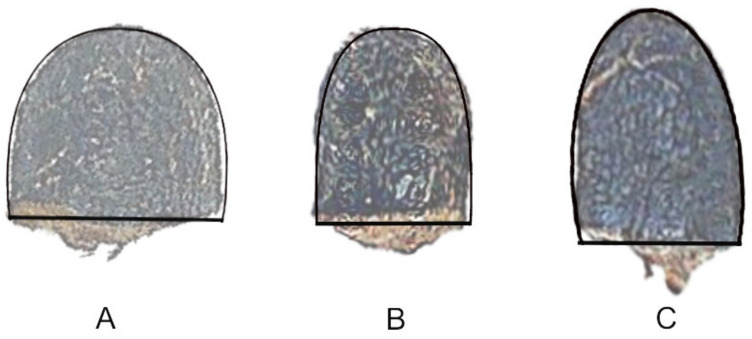
Seeds of *Rebutia heliosa* (**A**), *R. pseudodeminuta* (**B**) and *R. fiebrigii* (**C**) with their models superimposed.

Seeds of *Rebutia heliosa* Rausch, *R. pseudodeminuta* Backeb., and *R. fiebrigii* (Gürke) Britton & Rose are shown in Figure 1 with the corresponding models superimposed. These were derived from superellipses resulting from the representation of equation 1 with the values *p* = 2.3, a = 1.5, b = 2 for *R. heliosa*, *p* = 2.6, a = 1, b = 2.1 for *R. pseudodeminuta*, and *p* = 2.2, a = 1.5, b = 3 for *R. fiebrigii*.

The models for the seeds of three species of *Rebutia* are symmetric. The seed images of many other species are also symmetric, but in many cases, they present a certain degree of differentiation with a section straight corresponding to the hilum cup, and a rounded part in the opposite pole. This type of seeds is associated with a short funicle and were observed in species of *Echinopsis*, *Melocactus*, and *Thelocactus*.

For the seeds of *Echinopsis*, three models were identified for *E. calochlora*, *E. leucantha*, and *E. klingeriana*, obtained, respectively, from curves described by the following equations:

$$\left(y-\frac{10\sqrt{40-x^{2}}\;}{11}\right)\left(y+\frac{25\sqrt{40-x^{2}}\;}{22}+\frac{25}{50{\left(x/1.1{)}^{2}/30\right)}^{30}+8\;}\right)=0$$

$$\left(y-\frac{10\sqrt{40-x^{2}}\;}{11}\right)\left(y+\frac{25\sqrt{40-x^{2}}\;}{22}+\frac{25}{50{\left(x/1.1{)}^{2}/30\right)}^{30}+6\;}\right)=0$$

$$\left(y-\frac{10\sqrt{40-x^{2}}\;}{11}\right)\left(y+\frac{25\sqrt{40-x^{2}}\;}{22}+\frac{25}{50{\left(x/1.1{)}^{2}/30\right)}^{30}+4\;}\right)=0$$

**Figure 2:**
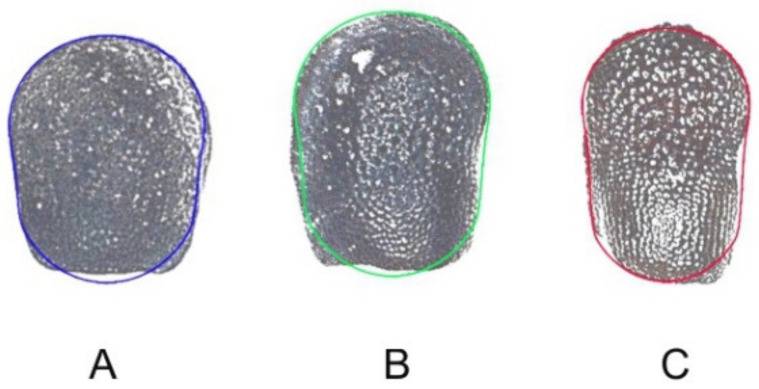
Seeds of Echinopsis species and their corresponding models. (**A**) *E. calochlora*; (**B**) *E. leucantha*; (**C**) *E. klingeriana*.

The three models are represented in Figure 2 with their corresponding seeds.

### 2.2. New Geometric Models Based on the Archimedean Spiral

**Figure 3:**
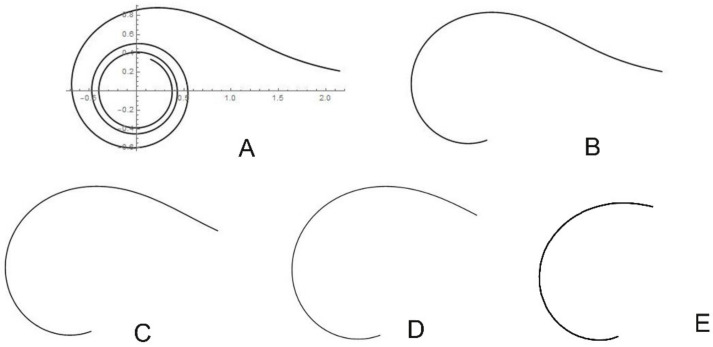
(**A**) Representation of the equation $r=t^{-\frac{1}{3}}$ for $t\in \left[0.1,\;20\right]$. (**B**–**E**) The equation represented for t in the intervals $\left[0.1,\;5\right]$, $\left[0.3,\;5\right]$, $\left[0.5,\;5\right]$, and $\left[1,\;5\right]$, respectively.

Figures resulting from the representations of the Archimedean (in wide sense) spiral $r=t^{-\frac{1}{3}}$ resemble the seed profiles of species in the Cactaceae. Figure 3 shows the geometric representation of the equation $r=t^{-\frac{1}{3}}$ with $t\in \left[0.1,\;20\right]$ (Figure 3A), $t\in \left[0.1,\;5\right]$ (Figure 3B), $t\in \left[0.3,\;5\right]$ (Figure 3C), $t\in \left[0.5,\;5\right]$ (Figure 3D), and $t\in \left[1,\;5\right]$ (Figure 3E).

**Figure 4:**
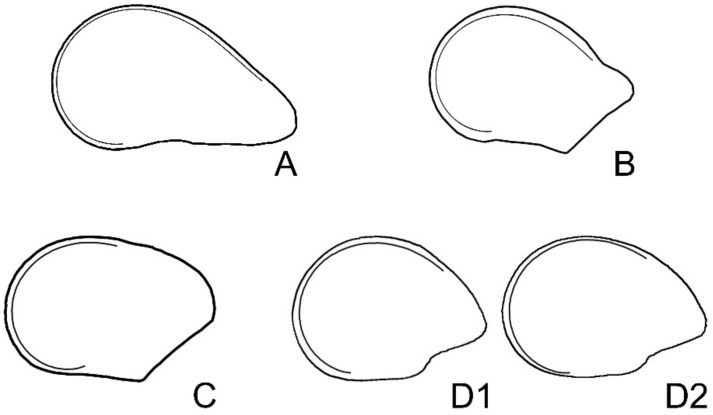
(**A**) Model HYLO1, designed for *Hylocereus undatus* seeds, with the curve corresponding to the equation $r=t^{-\frac{1}{3}}$ with $t\in \left[0.3,\;5\right]$ superimposed. (**B**) Model PACHY1, designed for *Pachycereus pringlei* seeds with the curve corresponding to the representation of equation $r=t^{-\frac{1}{3}}$ with $t\in \left[0.5,\;5\right]$ superimposed. (**C**) Model FERO1, designed for *Ferocactus herrerae*, and (**D1**,**D2**), Models ECHI1 and ECHI2, for *Echinocactus platyacanthus*, with the representation of equation $r=t^{-\frac{1}{3}}$ with $t\in \left[1,\;5\right]$. The complementary parts of the models (not represented in the curves) are obtained from the average silhouettes for each species in Corel Photo Paint.

From the curve in Figure 3C, we obtained model HYLO1, which adjusts to the shape of the upper part of *Hylocereus undatus* seeds. To compose the model (Figure 4A), Figure 3C was completed with the curve obtained from the average silhouette of seeds. Similarly, the curve in Figure 3D corresponds well to the upper part of the *Pachycereus pringlei* silhouette. To obtain PACHY1, the model for *Pachycereus pringlei* seeds, a curve is composed of Figure 3D and the average silhouette of seeds (Figure 4B). The model FERO1, for *Fereocactus herrerae*, was obtained with the curve represented in Figure 3E, which is completed by the corresponding average silhouette (Figure 4C). The two models for *Echinocactus platyacanthus*, ECHI1 and ECHI2, were composed of a dorsal part taken from the curve represented in Figure 3E, and a ventral part, corresponding to the border of the hilum cup, obtained from the average silhouette (Figure 4D1,D2). Models for *Opuntia* seeds were drawn based on the representation of the equation $r=t^{-1}+1-\cos\left(t\right)$ with $t\in \left[2,\;6\right]$; this equation was obtained as a modification of the cardioid $r=1-\cos\left(t\right)$ by the addition of the hyperbolic spiral $r=t^{-1}$.

### 2.3. Application of the Models to Seed Shape Quantification

Once a model was obtained, it was possible to use it for the analysis of seed lots and the comparison between populations or species. The following sections contain an overview of size and shape in diverse species and examples of the application of the models to seed shape quantification in seeds of *Hylocereus undatus*, *Pachycerus pringlei*, *Ferocactus herrerae, Echinocactus platyacanthus, Opuntia ficus-indica*, and *Pereskia bleo*.

#### 2.3.1. Overview of Seed Size and Shape in Some Cactus Species

The values obtained for area (A), perimeter (P), length (L), width (W), aspect ratio (L/W), circularity, and roundness in the seeds are shown in Table 1.

There were differences in size and shape between seeds of different species. The seeds of *F. herrerae*, *E. platyacanthus*, and *P. pringley* formed a group with smaller area values. The seeds of *Pereskia bleo* stand out as having higher area values, according to their reduced number of seeds per fruit (see later). The seeds of *H. undatus* and *P. bleo* had lower circularity. In respect to roundness, there were no statistical differences between *O. ficus-indica* and *E. platyacanthus* nor between *P. pringley* and *F. herrerae*. The differences between the values of circularity and roundness were due to irregularities in the seed surface, which increased the perimeter values, decreasing roundness. From the obtained data, it can be concluded that different models should be applied to each species. 

The values of the coefficient of variation comprised between 1.66 for circularity in *F. herrerae* and 12.84 for the same magnitude in *P. bleo* (Table 2).

Appendix A contains the box plots corresponding to four selected characters (area, aspect ratio, circularity, and roundness) of the six species under study.

#### 2.3.2. *Hylocereus undatus*

**Figure 5:**
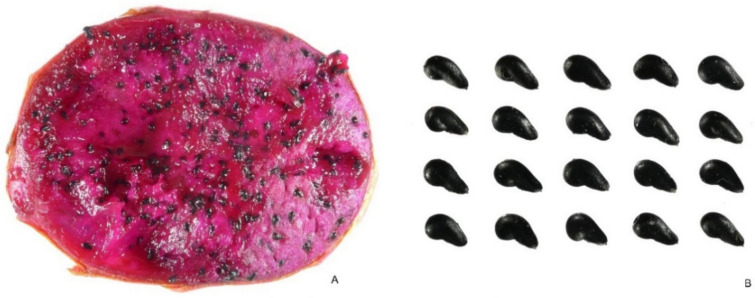
*Hylocereus undatus*. (**A**) Section of a fruit. (**B**) Seeds. Bar equals 1 cm.

Figure 5A shows the section of a fruit of H. undatus. Figure 5B presents 20 seeds obtained from this fruit. Mean J index of this group of seeds (with the corresponding model) was 90.5 (standard deviation equal to 2.5; maximum and minimum values are 98.5 and 81.1, respectively). The seeds of *H. undatus* adjusted well to the model. The coefficient of variation for the J index was 2.76.

#### 2.3.3. *Pachycereus pringlei*

**Figure 6:**
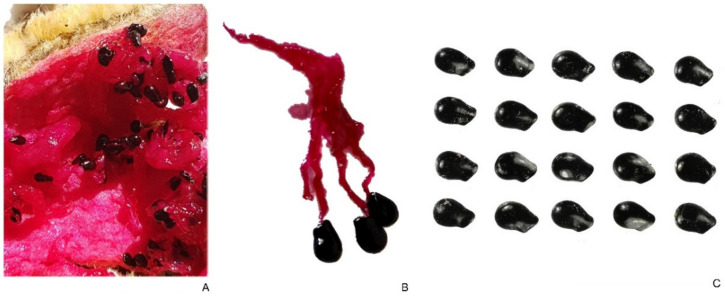
*Pachycereus pringlei*. (**A**) Seeds in a fruit. (**B**) Three seeds isolated with their funicles. (**C**) Seeds. Bar equals 1 cm.

Figure 6 shows the disposition of the seeds in the section of a fruit of *P. pringlei* and a detail of three seeds separated showing their funicles. Similar to *H. undatus,* the fruit is a fleshy berry and the seeds are free to develop without physical constrictions. The mean value of *J* index in a sample of 20 seeds (with the corresponding model) was 91.2 (standard deviation equal to 2.0; maximum and minimum values are 94.3 and 86.7, respectively). The seeds of *P. pringlei* adjusted well to the model. The coefficient of variation for the *J* index was 2.19.

#### 2.3.4. *Ferocactus herrerae*

**Figure 7:**
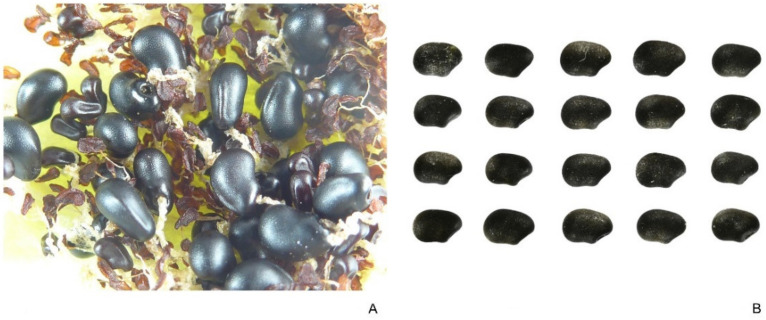
*Ferocactus herrerae*. (**A**) Seeds in a fruit. (**B**) Seeds. Bar equals 1 cm.

Figure 7A presents the distribution of seeds in a fruit and Figure 7B shows 20 seeds of *F. herrerae*. Mean value of *J* index in an observed group of 20 seeds (with the corresponding model) was 91.2 (standard deviation is 3.4; maximum and minimum values are 94.7 and 86.1, respectively). The seeds of *F. herrerae* adjusted well to the model. The coefficient of variation for the *J* index was 3.73.

#### 2.3.5. *Echinocactus platyacanthus*

**Figure 8:**
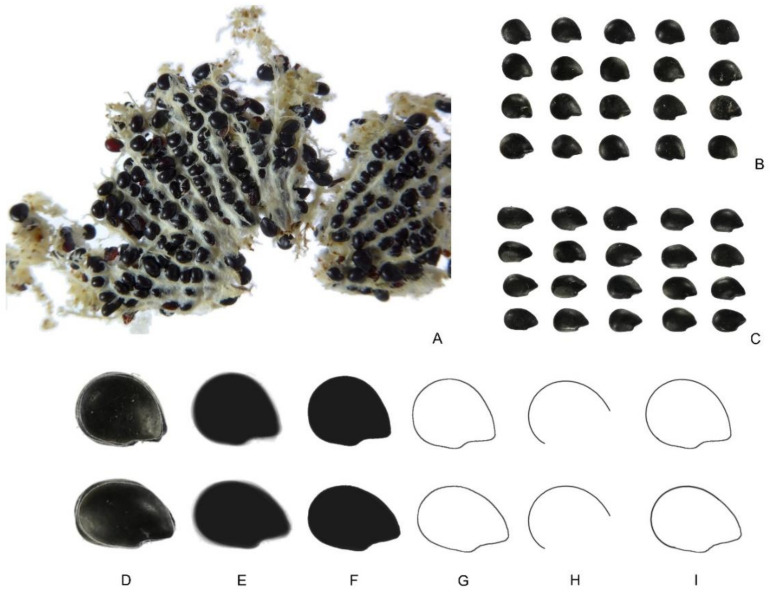
*Echinocactus platyacanthus*. (**A**) Seeds densely packaged in wool from an open fruit. The seeds have different orientations. (**B**) Short seeds; (**C**) Long seeds. Twenty seeds consisting of short (above) and long (below) types. (**D**,**E**,**F**) Successive steps to obtain the average silhouette. (**G**) Profile of the average silhouette. (**H**) Geometric curve used in the construction of the final model (**I**) for short (above) and long (below) seeds. Bars equal 1 cm.

Visual inspection of the seeds revealed more heterogeneity for shape in the seeds of *E. platyacanthus* than in the other species analyzed so far (*F. herrerae*, *P. pringlei*, and *H. undatus*). This is due to the disposition of seeds in the fruit, being densely packaged with different orientations (Figure 8A). Figure 8B,C show two groups of 20 seeds of *E. platyacanthus* formed, respectively, by short and long seeds. Measurements corresponding to *J* index values for the corresponding models are shown in Table 3. The coefficient of variation for the *J* index was 1.22 for short seeds and 1.62 for long seeds (standard deviations are 1.22 and 1.62, respectively).

#### 2.3.6. Cardioid-Derived Models for *Opuntia ficus-indica*

**Figure 9:**
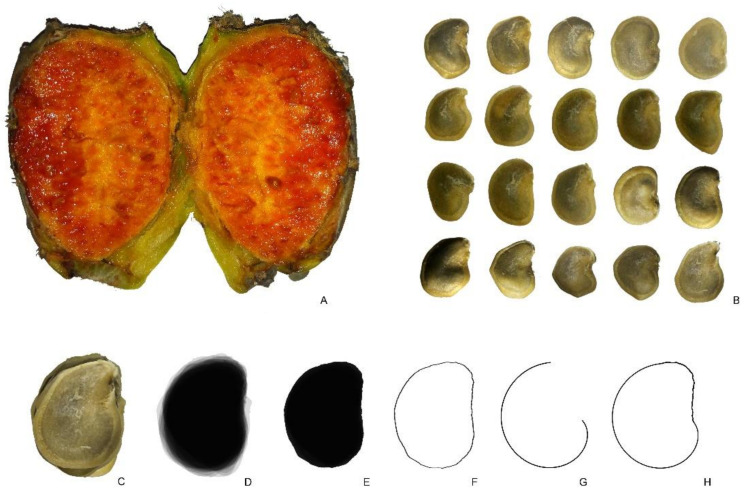
Seeds of *Opuntia ficus-indica*. (**A**) Open fruit. (**B**) Twenty seeds. (**C**) The images of twenty seeds were superimposed (**C**) and converted to their silhouettes (**D**) to obtain the average silhouette (**E**,**F**). (**G**) The geometric figure used as the basis for the model corresponds to the representation of the equation $r=t^{-1}+1-\cos\left(t\right)$ with $t\in \left[2,6\right]$. (**H**) Composed model with part of the average silhouette and part of the geometric figure. Bar equals 1 cm.

Figure 9 shows a sample of 20 seeds of *O. ficus-indica*. Mean value of *J* index in 20 seeds (with the model described in Section 4) was 90 (standard deviation is 2.3; maximum and minimum values are 94.7 and 86.1, respectively). The seeds of *O. ficus-indica* adjusted well to the model. The coefficient of variation for the *J* index was 2.52.

#### 2.3.7. Models for Species of *Pereskia* and *Maihuenia*

**Figure 10:**
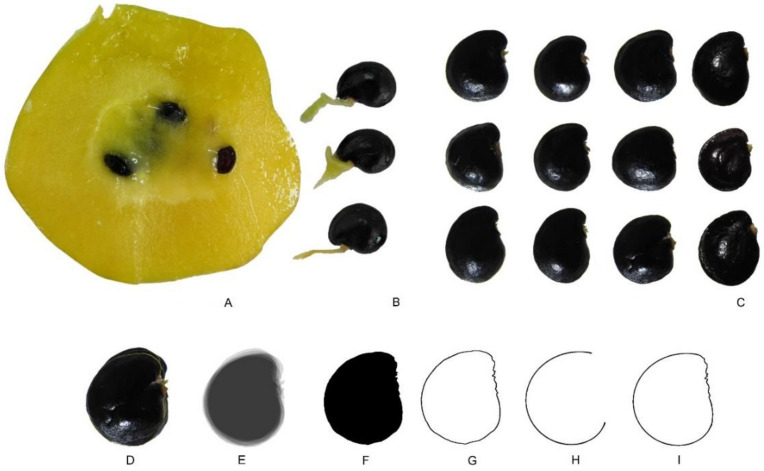
*Pereskia bleo*. (**A**) An open fruit with mature seeds. (**B**) Three seeds isolated with their funicles. (**C**) Twelve mature seeds. The obtention of the model is shown in the sequence from D to I. The images of nine seeds have been superimposed (**D**) and converted to their silhouettes (**E**) to obtain the average silhouette (**F**,**G**). (**H**) The curve used as the basis for the model corresponds to the equation $r=t^{-1/3}$ with $t\in \left[1,6\right]$, which was the basis for the design of the model. (**I**) Composed model with part of the average silhouette and part of the geometric figure. Bar equals 1 cm.

Geometric models derived from the Archimedean spiral defined well the silhouette of the seeds of *Pereskia* species. Figure 10 presents seed images of *Pereskia bleo* and the successive steps for the design of a model based on the representation of $r=t^{-1/3}$ with $t\in \left[2,6\right]$. Similar models based on the Archimedean spiral could be described for the seeds of *P. saccharosa* and *P. aculeata* as well as *Maihuenia poeppigii*. The mean value of the *J* index in a sample of 12 seeds (with the corresponding model) was 91.2 (standard deviation is 2.4; maximum and minimum values are 94.7 and 86.1, respectively). The seeds of *O. ficus-indica* adjusted well to the model. The coefficient of variation for the *J* index was 2.52.

## 3. Discussion

### 3.1. The Relationship between Seed Shape and Taxonomy

To explore seed shape in the Cactaceae, we have considered plant material belonging to the main subfamilies, with emphasis on the Cactoideae because it comprises about 80% of the total diversity [3]. A preliminary relationship between the models of seed morphology and the subfamilies of the Cactaceae is envisaged from the analysis presented. The overall morphology of the seeds in some species of *Maihuenia* and *Pereskia* (two basal subfamilies) shares a similarity with the general model described for *Opuntia* species in the Opuntioideae, based on fragments of an Archimedean spiral. Seed shape may share features between these three subfamilies with many plesiomorphic characters, such as the presence of leaves and shrub habit [38]. This general model could be considered as the basal shape morphology in seeds of this family that, during the cacti radiation, would be diversifying in different, more elongated shapes. The models described for species in the Cactoideae are based on longer fragments of the graphic representation of the same equation. 

The Cactoideae is the subfamily with the highest number of life forms, morphological types, and species richness [3,39], and here, we study species belonging to four of their tribes. The taxonomy of the Cactoideae is complex. Here, we have presented models for *Rebutia* (Trichocereae), *Hylocereus undatus* (Hylocereae), *Pachycereus pringlei* (Pachycereae), *Ferocactus herrerae*, and *Echinocactus platyacanthus* (Cacteae). The observation of seeds belonging to other species in these tribes suggests that there may be some correlation between seed shape of different species from the same tribe. For example, the seeds of *Disocactus nelsonii* (Britton & Rose) Linding, *Selenicereus grandiflorus* (L.) Britton & Rose, and *S. inermis* Britton & Rose (Hylocereae) have an overall shape similar to *Hylocerus undatus*. In addition, the species of the two genera belonging to the same tribe (*Ferocactus herrerae* and *Echinocactus platyacanthus*) had the same geometric figure as the basis for the respective models, and other and genera such as *Mammillaria* Haw. in the Cacteae share a similar form. The equations presented may be the basis for the design of models specific for tribes or genera, but this task requires the analysis of a number of seeds.

### 3.2. Seed Geometry in the Cactaceae

The seeds of the Cactaceae present an interesting diversity in shape. Forms that are frequent in other plant families, such as ovals and ellipses [33,40], are not that frequent in this family. Nevertheless, the seeds of some of their species can be described by their similarity to superellipses. These correspond to a group of symmetric seeds, represented by geometric figures with gradual transitions between them, such as the seeds of some species of *Rebutia* (*Aylostera*). A second group is represented by seeds whose shapes are also symmetric, but non-convex. These types of seeds are characteristic of some species in the Cactaceae and present a straight part in the hilum cup, and a rounded dorsal part, such as in species of *Echinopsis*, *Melocactus*, and *Thelocactus*, corresponding to the space occupied by the peripheral embryo [41,42].

Seeds in other species of the Cactaceae present characteristic shapes resembling the generalized Archimedean spirals with equation $r=t^{\alpha }$, see [43]. Seeds with spiral forms are found in species of *Coriaria* L., such as *C. arborea* Linds. and *C. japonica* A. Gray (Coriariaceae, Cucurbitales), as well as in *Sagittaria longiloba* Engelm. ex J.G. Sm. and *Echinodorus tenellus* (Mart. ex Schult.f.) Buchenau (Alismataceae, Alismatales) [23]. We are not aware of the developmental mechanism that may be responsible for seed shape in these species. In seeds of the Cactaceae, this shape is associated with the turn that the embryo makes during its development inside the fruit. In the case of *Opuntia* species, a turn is visible in *O. ficus-indica* seeds, where the funicle surrounds the ovule completely being like a thick third integument [44]. It is intriguing that the geometric figure resembling the silhouette of the seed in this species is the graphical representation of a sum of two functions corresponding, respectively, to the cardioid and to an Archimedean spiral. The first represents the growth from a fixed point; the second represents the progression of a point turning around a center, such as the embryo turns around itself during development [44]. Chapter 6 in D’Arcy Thompson’s book On Growth and Form [45] is dedicated to the Equiangular spiral, of general equation $r=a\;e^{-bt}.$ It gives many examples of spiral forms in nature including in plants, such as in the helicoid cymes, but it does not offer any example from seeds.

The reniform shape, better defined as the cardioid curve, is also discussed by D’Arcy Thompson in chapter 9 of his book [45]. This chapter is entitled “On the theory of transformations or the comparison of related forms”, and it begins by differentiating between descriptive and analytical languages. The term reniform belongs to the former, while the expression “cardioid curve”, belongs to the latter, because it expresses the geometric representation of an algebraic identity and, as such, it permits to study and interpret possible interactions and allows quantification. The chapter makes emphasis on the formal analysis of the shapes of the natural objects, relating them with the movements that are active during their formation. A section of it refers to the shapes of leaves as representations of polar equations, with a “point of arrest” of zero growth in a direction given, and growth of maximum velocity towards the leaf tip in the opposite direction. This corresponds to the cardioid curve, a shape that is common in leaves and seeds [23,24,25,26,27,28].

The molecular basis of the formation of developmental patterns acquired a great impulse with the application of genomics and the analysis of mutants in *Arabidopsis*. The formation of phyllotactic spirals in *Arabidopsis*, similar to the development of the inflorescences in the cauliflower, have revealed a complex set of gene interactions [46]. The shapes of seeds are at the interphase between the developmental mechanisms of the seed and the embryo regulated at the molecular level and the ecological processes of dispersion. In many instances, the seeds of cacti have complex adaptations to diverse types of zoochory associated with water transport that could be variable between related species. However, in the subfamily Opuntioideae (~250 species), the hard bony arils present in all the species except in the genus *Pterocactus* [3] are key diagnostic characters that are very useful in taxonomy. By studying the shape of seeds in a larger number of species and genera, we may confirm whether their general shape remains constant within each tribe, and thus, they can also be considered as a taxonomic character in this botanical family.

## 4. Materials and Methods

### 4.1. Plant Material

To explore seed shape, plant species belonging to the main subfamilies, with emphasis on the most diverse Cactoideae, have been considered. The seeds of *Echinocactus platyacanthus* Link & Otto and *Ferocactus herrerae* J.G.Ortega were obtained from plants grown in the campus of University of Alicante (San Vicent del Raspeig, Alicante, Spain); the seeds of *Pachycereus pringlei* (S.Watson) Britton & Rose were from a fruit grown in El Huerto del Cura (Elche, Alicante, Spain); and the seeds of *Hylocereus undatus* (Haworth) D.R. Hunt were obtained from a commercial pitahaya bought on a local market in Albir (Alicante, Spain). All the fruits mentioned above were collected between 6 August and 12 August 2021, and their seeds photographed in September, 2021. Opuntia *ficus-indica* (L.) Mill seeds are from plants grown in Ambato, Tungurahua (Ecuador) and Villar de Peralonso, Salamanca (Spain) collected in 2020. *Pereskia bleo* (Kunth) DC seeds were collected in a public garden close to the Malecón Boayaku at Puyo (Pastaza, Ecuador) on 12 October 2021 and photographed in the next days. After being photographed, the seeds were returned to their origin. A sample of this plant will be deposited in the Amazon State University Herbarium (ECUAMZ).

The images of seeds of *Rebutia* K. Schum., *Echinopsis* Zucc., *Maihuenia* (Phil. ex F.A.C.Weber) K. Schum., and *Thelocactus* Britton & Rose were obtained from https://cactus-aventures.com/TaxonomySeedGallery/Seeds%20album/ (accessed on 27 September 2021). On this webpage, *Rebutia* species are shown with the name *Aylostera*, which is a synonym. 

### 4.2. Photography and Image Analysis

Photographs were taken with a camera Z6 with an objective AF-S Micro NIKKOR 60 mm f/2.8G ED. Composed images containing 10–30 seeds per accession were prepared with Corel Photo Paint. For *O. ficus indica* and *P. bleo*, which were collected in Ecuador, the photographs were taken with a camera Cannon PowerShot SX520 HS.

Values of the area (A), perimeter (P), length (L), width (W), aspect ratio (AR is equal to L/W), circularity (C), and roundness (R) of the seed images were obtained in Image J [36].

### 4.3. Obtention of an Average Silhouette for Each Group of Seeds

The average silhouette is a representative image of seed shape for each group of seeds. It was obtained in Corel Photo Paint, by the protocol described in [37] (a detailed video is available at Zenodo: https://zenodo.org/record/4478344#.YBPOguhKiM8, accessed on 9 November 2021). The layers containing the seeds were superimposed and the opacity is given a value of 30 in all layers. All the layers were combined, and the brightness is adjusted to a minimum value. From this image, we are interested in the inner region representing the area where most of the seeds coincide, which is the darkest area. To select it, we use the magic wand tool, and with a tolerance equal to 10, this selection is copied and pasted as a new layer. Twenty seeds were used to obtain the average silhouettes for *Echinocactus platyacanthus*, *Ferocactus herrerae*, *Hylocereus undatus*, *Opuntia ficus-indica*, and *Pachycereus pringley*. For *Echinocactus platyacanthus*, the average silhouettes were obtained separately from 20 seeds of long and short seeds. For *Pereskia*, average silhouettes were obtained from 9 seeds.

### 4.4. Geometric Models

#### 4.4.1. Geometric Models for the Symmetric Seeds of *Rebutia* and *Echinopsis*

Two types of models were defined for the symmetric seeds: the first is composed by superellipses, and the second is derived from the combination of two semi-ellipses.

Superellipses correspond to equations of the form:

$${\left|\frac{x}{a}\right|}^{p}+{\left|\frac{y}{b}\right|}^{p}=1$$

With p>0, and they are similar to models presented for the Arecaceae [34].

A second group of models applied to the symmetric seeds of the Cactaceae is derived from the equation of an ellipse with semi-axes 1 and 1/b, b>0, written here as:

$$\left(\sqrt{1-x^{2}}-b\;y\right)\left(\sqrt{1-x^{2}}+b\;y\right)=0$$

To allow the modification of each semi-ellipse separately, as was done for the Vitaceae [35].

#### 4.4.2. Geometric Models for the Asymmetric Seeds of *Echinocactus*, *Ferocactus*, *Hylocereus*, *Opuntia ficus-indica*, *Pachycereus*, and *Pereskia*

The models were based on the Archimedean spiral with equation, in the following polar coordinates:

$$r=t^{-\frac{1}{3}}$$

where t, r stand for the polar angle and radius, respectively.

Graphic representations of the equations were obtained with Mathematica, and the curves were superimposed in Corel Photo Paint to seed images to verify and quantify the coincidence of shape. Mixed models were composed with a part corresponding to the geometric representation of an algebraic equation and the other obtained from average silhouettes (Figure 1). 

#### 4.4.3. Drawing of the Models

**Figure 11:**
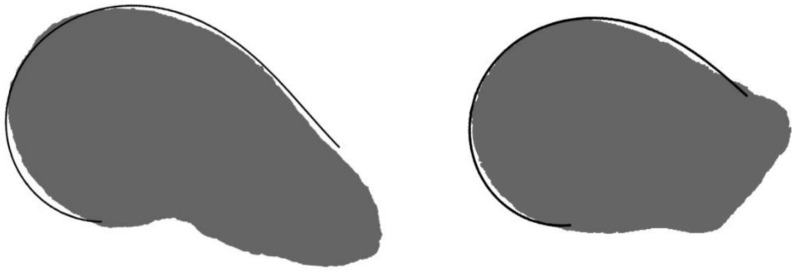
Graphic representation of the method used to draw the model in *Hylocereus undatus* and *Pachycerus pringlei* seeds. The curve adjusting to the seed profile is superimposed to the average silhouette. The fragment of the seed image uncovered by the curve is completed with the corresponding fragment of the average silhouette with Corel Photo Paint.

The geometric models were superimposed to the average silhouettes for each species searching the best fit between bot images and the fragment of the silhouette uncovered by the model was completed with the corresponding fragment of the average silhouette (Figure 11).

### 4.5. Seed Shape Quantification and Testing of the Models: J Index

**Figure 12:**
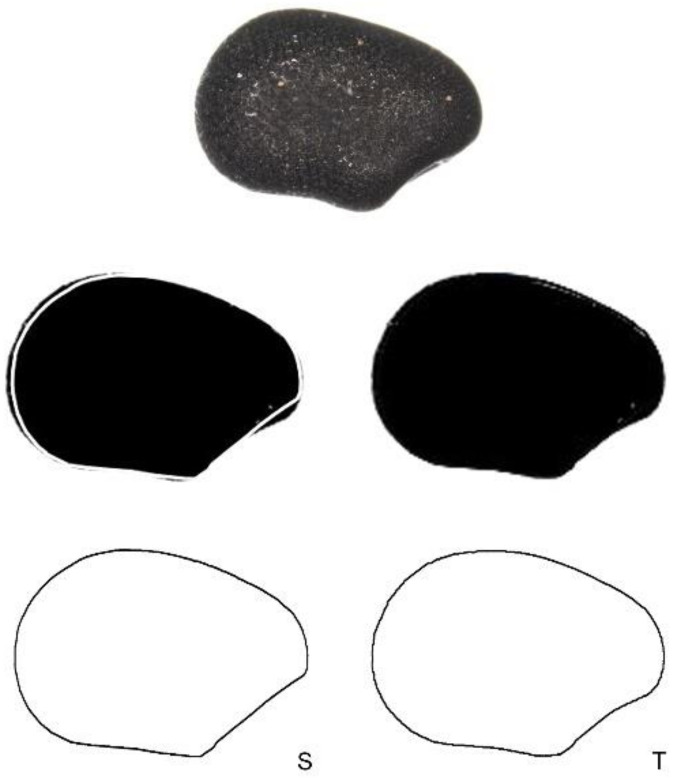
Graphic representation of the method used to calculate the *J* index. Top: image of a seed of *Ferocactus herrerae*. Middle: the same image in black with the model superimposed; left: the model in white to obtain the shared area (**S**); right: the model in black to obtain total area (**T**). Below: Interpretation of the images by ImageJ. The values for the estimated areas are 294.241 pixel for the shared (**S**) and 320.870 pixel for the total area (**T**), respectively. The *J* index of this example equals to 91.2.

The *J* index is a measurement of the similarity between a bi-dimensional image of a seed and the geometric model. The *J* index is the percentage of the area that is shared by both images once they are superimposed for maximum similarity. It is quantified as:

J=S/T×100

where S is the area shared between the seed and the model and T is the total area occupied by both figures. The *J* index ranges between 0 and 100, reaching maximum values when the geometric model and the seed image areas coincide. High scores on the *J* index reflect similarity of a seed image with a given model, meaning that the model provides a precise definition of seed shape for a particular species. A good adjustment to the model is considered when *J* index values are superior to 90 [47]. Figure 12 shows an example of the images used for J index quantification.

### 4.6. Statistical Analysis

Mean, minimum, and maximum values and the standard deviation were obtained for area (A), perimeter (P), length (L), width (W), aspect ratio (AR is equal to L/W), circularity (C), and roundness (R) as well as the *J* index with the different models. Statistics were obtained using IBM SPSS statistics v25 (SPSS 2017) and R. software v. 4.0.5 (R core team 2020 [48]. According to the Kolmogorov and Shapiro–Wilk tests, it cannot be rejected that the data came from a normally distributed population. A one-way ANOVA was used to infer significant differences between species for the measured variables, followed by Scheffé post-hoc tests to provide specific information on which means were significantly different from one another. *p* values less than 0.05 were considered significant. The coefficient of variation was calculated as CV_trait_ = standard deviation_trait_/mean_trait_ × 100 [49].

## 5. Conclusions

New models based on geometric figures are proposed for seed shape description and quantification in species of the Cactaceae. Models derived from equation $r=t^{-\frac{1}{3}}$ were applied to the seeds of *Echinocactus*, *Ferocactus*, *Hylocereus*, *Pachycereus*, and *Pereskia*. The seeds of *Opuntia ficus-indica* adjust well to a model derived from equation $r=t^{-1}+1-\cos\left(t\right).$ Models for *Rebutia* species were based on the equations for superellipses, while for *Echinopsis*, the models derived from the representation of two semi-ellipses. The comparison with geometric models is proposed as a basis for seed description and quantification in the Cactaceae.

## Figures and Tables

**Table 1:** Area (A), perimeter (P), length (L), width (W), aspect ratio (AR is L/W), circularity (C), and roundness (R) in seed samples of six species belonging to different subfamilies of the Cactaceae. A is given in mm^2^, P, L, and W in mm. The mean values marked with the same superscript letter in each column do not differ significantly at *p* < 0.05 (Scheffé’s test). Standard deviation values are given in parentheses. The second row of each cell shows minimum/maximum values for each data entry. N indicates the number of seeds analyzed.

	N	A (mm^2^)	P (mm)	L (mm)	W (mm)	AR	C	R
*Echinocactus platyacanthus*	153	2.97 ^a^ (0.15) 2.50/3.42	6.83 ^b^ (0.26) 6.29/8.05	2.23 ^a^ (0.09) 2.03/2.54	1.70 ^b^ (0.07) 1.50/1.87	1.32 ^b^ (0.08) 1.17/1.54	0.80 ^cd^ (0.04) 0.60/0.86	0.76 ^c^ (0.05) 0.65/0.86
*Ferocactus herrerae*	121	2.54 ^a^ (0.20) 1.72/3.02	6.19 ^a^ (0.25) 5.09/6.73	2.16 ^a^ (0.09) 1.73/2.36	1.49 ^a^ (0.06) 1.27/1.63	1.45 ^c^ (0.04) 1.36/1.63	0.83 ^d^ (0.01) 0.77/0.86	0.69 ^b^ (0.02) 0.61/0.73
*Hylocereus undatus*	121	3.04 ^a^ (0.18) 2.53/3.56	7.46 ^c^ (0.38) 6.75/9.55	2.69 ^b^ (0.12) 2.33/2.96	1.44 ^a^ (0.06) 1.30/1.59	1.87 ^d^ (0.11) 1.62/2.17	0.69 ^a^ (0.05) 0.43/0.75	0.54 ^a^ (0.03) 0.46/0.62
*Opuntia ficus-indica*	117	12.34 ^c^ (1.41) 8.71/15.70	14.49 ^e^ (1.11) 12.34/19.92	4.57 ^d^ (0.37) 3.71/5.63	3.43 ^d^ (0.25) 2.82/4.00	1.34 ^b^ (0.14) 1.07/1.74	0.74 ^b^ (0.07) 0.41/0.84	0.76 ^c^ (0.08) 0.57/0.94
*Pachycereus pringley*	151	5.95 ^b^ (0.49) 4.25/7.04	9.89 ^d^ (0.63) 8.37/12.82	3.37 ^c^ (0.15) 2.95/3.72	2.25 ^c^ (0.11) 1.83/2.46	1.50 ^c^ (0.07) 1.30/1.72	0.77 ^bc^ (0.06) 0.49/0.83	0.67 ^b^ (0.03) 0.58/0.77
*Pereskia bleo*	13	39.61 ^d^ (3.83) 31.38/44.78	26.78 ^f^ (2.23) 22.38/29.93	7.81 ^e^ (0.51) 6.92/8.60	6.45 ^e^ (0.36) 5.77/7.05	1.21 ^a^ (0.08) 1.04/1.34	0.70 ^a^ (0.09) 0.53/0.83	0.83 ^d^ (0.06) 0.74/0.96

**Table 2:** Values of the coefficient of variation for area (CV(A)), perimeter (CV(P)), length (CV(L)), width (CV(W)), aspect ratio (CV(AR)), circularity (CV(C)), and roundness (CV(R)) in seed samples of six species belonging to different subfamilies of the Cactaceae. N indicates the number of seeds analyzed.

	N	CV(A)	CV(P)	CV(L)	CV(W)	CV(AR)	CV(C)	CV(R)
*Echinocactus platyacanthus*	153	5.13	3.85	3.92	4.01	6.11	5.19	5.97
*Ferocactus herrerae*	121	7.87	4.05	4.12	4.20	2.49	1.66	2.47
*Hylocereus undatus*	121	5.84	5.14	4.43	3.87	5.80	7.41	6.06
*Opuntia ficus-indica*	117	11.39	7.63	8.16	7.24	10.67	9.87	10.49
*Pachycereus pringley*	151	8.22	6.42	4.42	4.97	4.44	7.30	4.48
*Pereskia bleo*	13	9.67	8.33	6.47	5.59	6.64	12.84	6.97

**Table 3:** Values of area, perimeter, length, width, aspect ratio, circularity, and roundness in two groups of short (S) and long (L) seeds in *E. platyacanthus*. *J* index SM and *J* index LM are the values of *J* index obtained with the short and long models, respectively. The mean values marked with the same superscript letter in each column do not differ significantly at *p* < 0.05 (Scheffé’s test). N indicates the number of seeds analyzed.

	N	Area	P	L	W	AR	C	R	*J* Index SM	*J* Index LM
**S**	20	2.94 ^a^ (0.15) 2.65/3.21	6.69 ^a^ (0.21) 6.34/7.07	2.15 ^a^ (0.06) 2.05/2.28	1.74 ^b^ (0.05) 1.62/1.80	1.24 ^a^ (0.04) 1.18/1.30	0.83 ^b^ (0.02) 0.80/0.86	0.81 ^b^ (0.02) 0.77/0.85	92.1 ^b^ (1.12) 88.9/93.6	87.5 ^a^ (1.36) 84.8/90.3
**L**	20	2.99 ^a^ (0.14) 2.74/3.19	6.86 ^b^ (0.19) 6.56/7.25	2.36 ^b^ (0.07) 2.24/2.50	1.61 ^a^ (0.05) 1.48/1.69	1.46 ^b^ (0.06) 1.37/1.60	0.80 ^a^ (0.02) 0.75/0.83	0.68 ^a^ (0.03) 0.63/0.73	87.7 ^a^ (1.90) 83.7/90.6	91.7 ^b^ (1.49) 88.9/93.8

**Figure A1:**
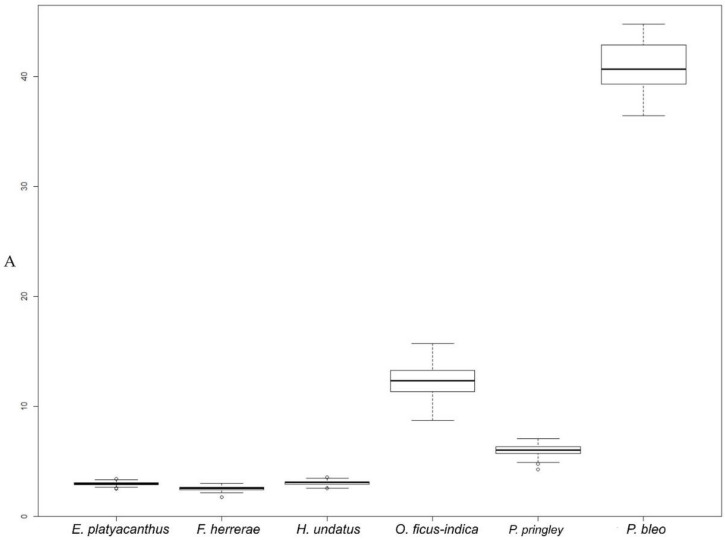
Values of area for seeds of the species *Echinocactus platyacanthus*, *Ferocactus herrerae*, *Hylocereus undatus*, *Opuntia ficus-indica*, *Pachycereus pringley*, and *Pereskia bleo*.

**Figure A2:**
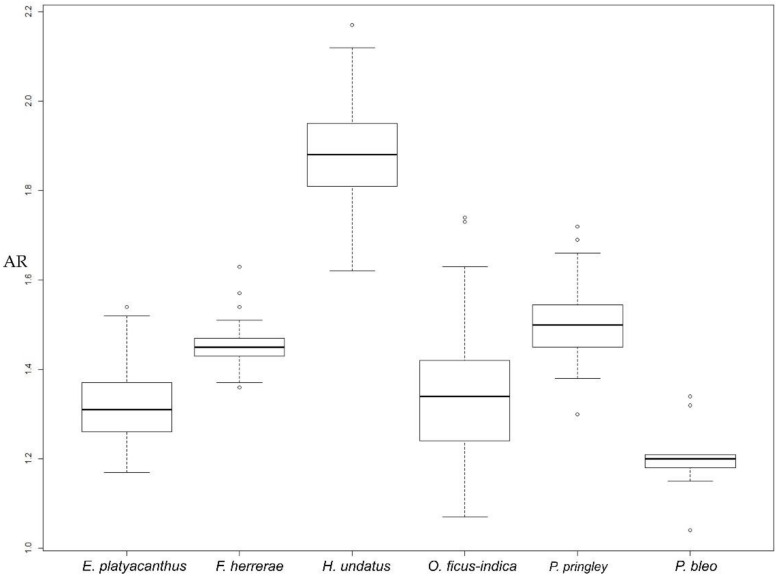
Values of aspect ratio for seeds of the species *Echinocactus platyacanthus*, *Ferocactus herrerae*, *Hylocereus undatus*, *Opuntia ficus-indica*, *Pachycereus pringley*, and *Pereskia bleo*.

**Figure A3:**
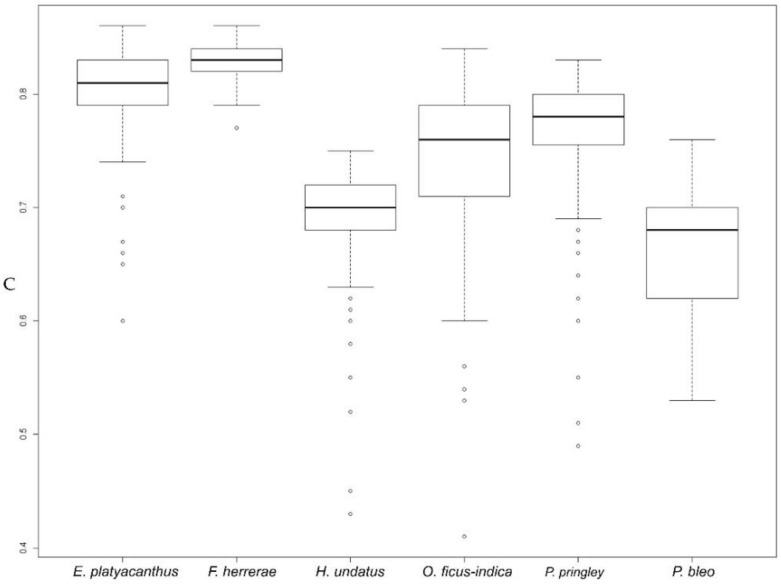
Values of circularity index for seeds of the species *Echinocactus platyacanthus*, *Ferocactus herrerae*, *Hylocereus undatus*, *Opuntia ficus-indica*, *Pachycereus pringley*, and *Pereskia bleo*.

**Figure A4:**
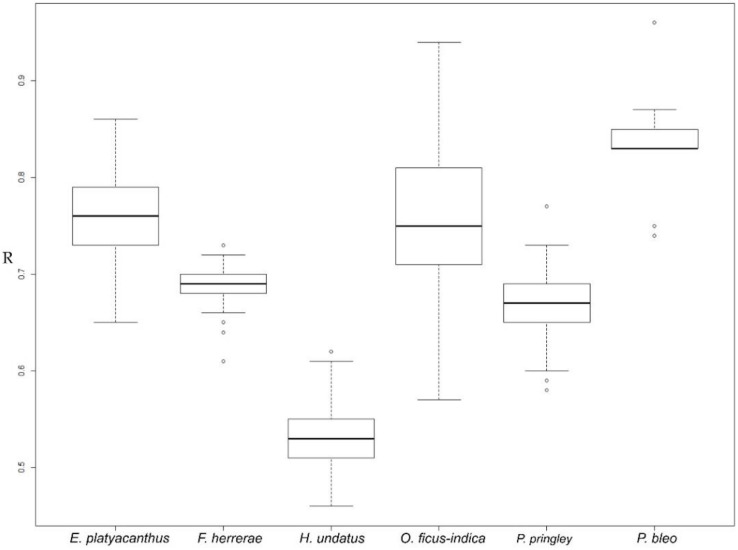
Values of roundness for seeds of the species *Echinocactus platyacanthus*, *Ferocactus herrerae*, *Hylocereus undatus*, *Opuntia ficus-indica*, *Pachycereus pringley*, and *Pereskia bleo*.

## Data Availability

The data presented in this study are available in article.

## Appendix A

Box plot representations can be found below, corresponding to the measurements (area, aspect ratio, circularity, and roundness) for the species *Echinocactus platyacanthus*, *Ferocactus herrerae*, *Hylocereus undatus*, *Opuntia ficus-indica*, *Pachycereus pringley*, and *Pereskia bleo*.
